# Receptor-Interacting Protein Kinase-2 Inhibition by CYLD Impairs Antibacterial Immune Responses in Macrophages

**DOI:** 10.3389/fimmu.2015.00650

**Published:** 2016-01-19

**Authors:** Katharina Wex, Ursula Schmid, Sissy Just, Xu Wang, Rebecca Wurm, Michael Naumann, Dirk Schlüter, Gopala Nishanth

**Affiliations:** ^1^Institute of Medical Microbiology and Hospital Hygiene, Otto-von-Guericke University Magdeburg, Magdeburg, Germany; ^2^Institute of Experimental Internal Medicine, Otto-von-Guericke University Magdeburg, Magdeburg, Germany; ^3^Organ-Specific Immune Regulation, Helmholtz Centre for Infection Research, Braunschweig, Germany

**Keywords:** *Listeria monocytogenes*, receptor-interacting protein kinase-2, CYLD, macrophages, antibacterial immune responses

## Abstract

Upon infection with intracellular bacteria, nucleotide oligomerization domain protein 2 recognizes bacterial muramyl dipeptide and binds, subsequently, to receptor-interacting serine/threonine kinase 2 (RIPK2), which activates immune responses via the nuclear factor kappa-light-chain enhancer of activated B cells (NF-κB) and extracellular signal-regulated kinase (ERK) pathways. Activation of RIPK2 depends on its K63 ubiquitination by E3 ligases, whereas the deubiquitinating enzyme A20 counter regulates RIPK2 activity by cleaving K63-polyubiquitin chains from RIPK2. Here, we newly identify the deubiquitinating enzyme CYLD as a new inhibitor of RIPK2. We show that CYLD binds to and removes K63-polyubiquitin chains from RIPK2 in *Listeria monocytogenes* (Lm) infected murine bone marrow-derived macrophages. CYLD-mediated K63 deubiquitination of RIPK2 resulted in an impaired activation of both NF-κB and ERK1/2 pathways, reduced production of proinflammatory cytokines interleukin-6 (IL-6), IL-12, anti-listerial reactive oxygen species (ROS) and nitric oxide (NO), and, finally, impaired pathogen control. In turn, RIPK2 inhibition by siRNA prevented activation of NF-κB and ERK1/2 and completely abolished the protective effect of CYLD deficiency with respect to the production of IL-6, NO, ROS, and pathogen control. Noteworthy, CYLD also inhibited autophagy of *Listeria* in a RIPK2-ERK1/2-dependent manner. The protective function of CYLD deficiency was dependent on interferon gamma (IFN-γ) prestimulation of infected macrophages. Interestingly, the reduced NF-κB activation in CYLD-expressing macrophages limited the protective effect of IFN-γ by reducing NF-κB-dependent signal transducers and activators of transcription-1 (STAT1) activation. Taken together, our study identifies CYLD as an important inhibitor of RIPK2-dependent antibacterial immune responses in macrophages.

## Introduction

Macrophages play a crucial role in the innate immune response including sensing and phagocytosis of bacterial pathogens at the site of infection (1). In bacterial infections, phagocyte activation is augmented by type II interferon (IFN-γ) produced by natural killer (NK) and T cells (2). IFN-γ primarily signals through the Janus kinase (JAK) – signal tranducers and activators of transcription (STAT)-1 pathway and primes the secretion of proinflammatory cytokines, production of superoxide anions, generation of nitrogen and oxygen free radicals and thereby conditions macrophages as potent antibacterial effector cells (3, 4). Of note, STAT1 is also activated by type I IFNs, which can be produced by various cell types including macrophages in bacterial infection (5). However, in contrast to IFN-γ, type I IFN is not protective and may even exacerbate infection with *Listeria monocytogenes* (Lm), a facultative intracellular bacterium (6, 7).

In addition to cell surface and endosomal Toll-like receptors, cytosolic nucleotide oligomerization domain protein (NOD)-like receptors, in particular NOD2, recognize intracellular bacteria such as Lm. Activated NOD2 binds to receptor-interacting serine/threonine kinase 2 (RIPK2) (8). RIPK2 undergoes K63 polyubiquitination resulting in the activation of nuclear factor kappa-light-chain enhancer of activated B cells (NF-κB) and mitogen-activated protein kinases (MAPKs) (9–13). The activity of RIPK2 is counter-regulated by the deubiquitinating enzyme A20, which disassembles K63-linked ubiquitin chains from RIPK2 (10). NOD2-dependent RIPK2 activation is essential for an effective antibacterial response, since RIPK2-deficient macrophages have an impaired pathogen control (14–16) and RIPK2-deficient mice are highly susceptible to listeriosis (17).

*Listeria monocytogenes* may cause severe infections in pregnant women and immunocompromised patients (18). In mice, *Listeria* rapidly home to Kupffer cells, i.e., liver-resident macrophages (19). In addition, uninfected myeloid cell populations including macrophages, monocytes, and TNF/iNOS-producing dendritic cells are recruited to the site of infection and are crucial for the control of listeriosis (20). In macrophages, both NF-κB and MAPK induce the synthesis of protective (i) proinflammatory cytokines including IL-6 and IL-12, (ii) ROS, (iii) nitric oxide (NO), and (iv) autophagosomes (21–24). The formation of autophagosomes is either induced via the NOD2/RIPK2/extracellular signal-regulated kinase (ERK) pathway (25) or NOD2 independent by the autophagy-related protein 16-1 (ATG16L1) (26). In the autophagosomes, phagocytosed Lm are killed by ROS (27–29). In further amplification of antibacterial activity, ROS activates the STAT1 pathway leading to increased production of NO, which reacts with ROS to produce peroxynitrite, an even stronger antibacterial molecule (30).

The deubiquitinating enzyme CYLD cleaves K63-linked polyubiquitin chains from specific substrates (31), including tumor necrosis factor receptor-associated factors (TRAF)-2, TRAF6, transforming growth factor beta-activated kinase 1 (TAK1), B cell lymphoma 3 (BCL3), STAT3, nuclear factor kappa B essential modulator (NEMO), and retinoic acid inducible gene 1 (RIG-1) (32–36), and negatively regulates the activation of NF-κB, MAPKs, and type I IFN production. In infectious diseases, CYLD has a disease-specific effect and CYLD-deficient mice suffer from exacerbated *Escherichia coli* pneumonia and *Haemophilus influenzae* middle ear infection (37, 38) but are protected against lethal *Streptococcus pneumoniae* and Lm infection (36, 37).

Earlier, we have shown that macrophage-derived IL-6 induced STAT3-dependent protective fibrin production by hepatocytes in listeriosis (36). However, it remained unclear whether CYLD also directly regulated the antibacterial function of infected macrophages, in particular, the NOD2-dependent activation of NF-κB, MAPKs, and autophagy. Here, we newly identified that CYLD inhibits NOD2-dependent antibacterial activity of macrophages by K63 deubiquitination of RIPK2.

## Results

### CYLD Impairs Production of Cytokines, ROS, and NO and Reduces Activation of NF-κB, ERK1/2, and p38MAPK in Lm-Infected BMDM

**Figure 1:**
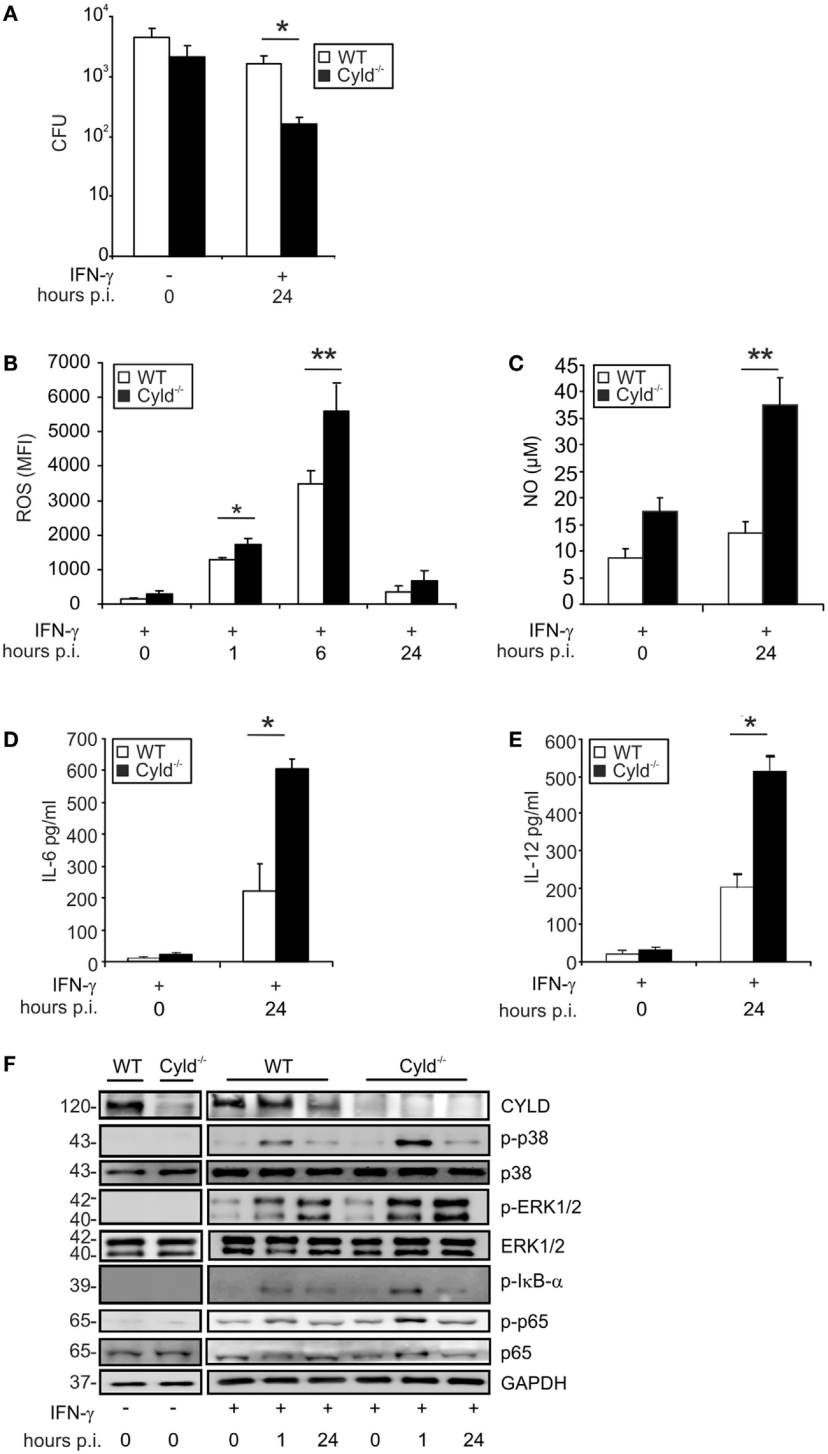
**CYLD impairs pathogen control and decreases cytokine production and activation of NF-κB and MAP kinases**. WT and Cyld^−/−^ BMDM (1 × 10^6^cells/group), stimulated with IFN-γ (100 U/ml) for 24 h followed by infection with Lm (MOI 5:1). **(A)** The bacterial load was determined in 1 × 10^6^ IFN-γ-stimulated and unstimulated Lm-infected WT and Cyld^−/−^ BMDM 24 h p.i. **(B)** Levels of intracellular ROS were analyzed by flow cytometry in 1 × 10^6^ uninfected (0 h p.i.) and infected (1, 6, 24 h p.i.) WT and Cyld^−/−^ BMDM. **(C)** The amount of NO was measured in the supernatant of 1 × 10^6^ uninfected and infected WT and Cyld^−/−^ BMDM colorimetrically using a Griess assay kit. **(D,E)** IL-6 **(D)** and IL-12 **(E)** were determined in the supernatant of 1 × 10^6^ Lm-infected WT and Cyld^−/−^ BMDMs by flow cytometric bead array. **(F)** Proteins were isolated from 1 × 10^6^ uninfected and infected WT and Cyld^−/−^ BMDM at the indicated time points and stained with antibodies specific for CYLD, p-p38, p38, p-ERK1/2, ERK1/2, p-IκB-α, p-p65, and p65. GAPDH was used as loading control. In **(A–E)**, data represent the mean + SD from triplicate wells. In **(A–F)**, data from one of the three representative experiments are shown.

Upon infection with Lm, Cyld^−/−^ BMDM showed no significant improvement in pathogen control as compared to WT BMDM (Figure 1A, *p* < 0.05). In good agreement with previous reports (39, 40), pretreatment of BMDM with IFN-γ, greatly augmented the listericidal activity of BMDM. Importantly, the IFN-γ-induced listericidal effect was significantly stronger in Cyld^−/−^ as compared to WT BMDM. Since CYLD only regulated pathogen control upon IFN-γ stimulation, we further focused on the function of CYLD in IFN-γ-stimulated Lm-infected BMDM.

In addition to pathogen control, the production of listericidal ROS and NO as well as the cytokines IL-6 and IL-12 were significantly increased in IFN-γ-treated Lm-infected Cyld^−/−^ BMDM (Figures 1B–E). To identify the underlying CYLD-regulated signaling pathways, the activation of NF-κB, ERK1/2, and p38MAPK were analyzed by WB in IFN-γ-stimulated Lm-infected BMDM. As displayed in Figure 1F, Cyld^−/−^ BMDM showed enhanced activation of NF-κB as indicated by increased phosporylation of p65 and IκB-α. In addition, activation of ERK1/2 and p38MAPK were augmented in Cyld^−/−^ BMDM. In contrast, stimulation with IFN-γ only was insufficient to induce activation of these signaling pathways.

### CYLD Impairs Activation of STAT1

**Figure 2:**
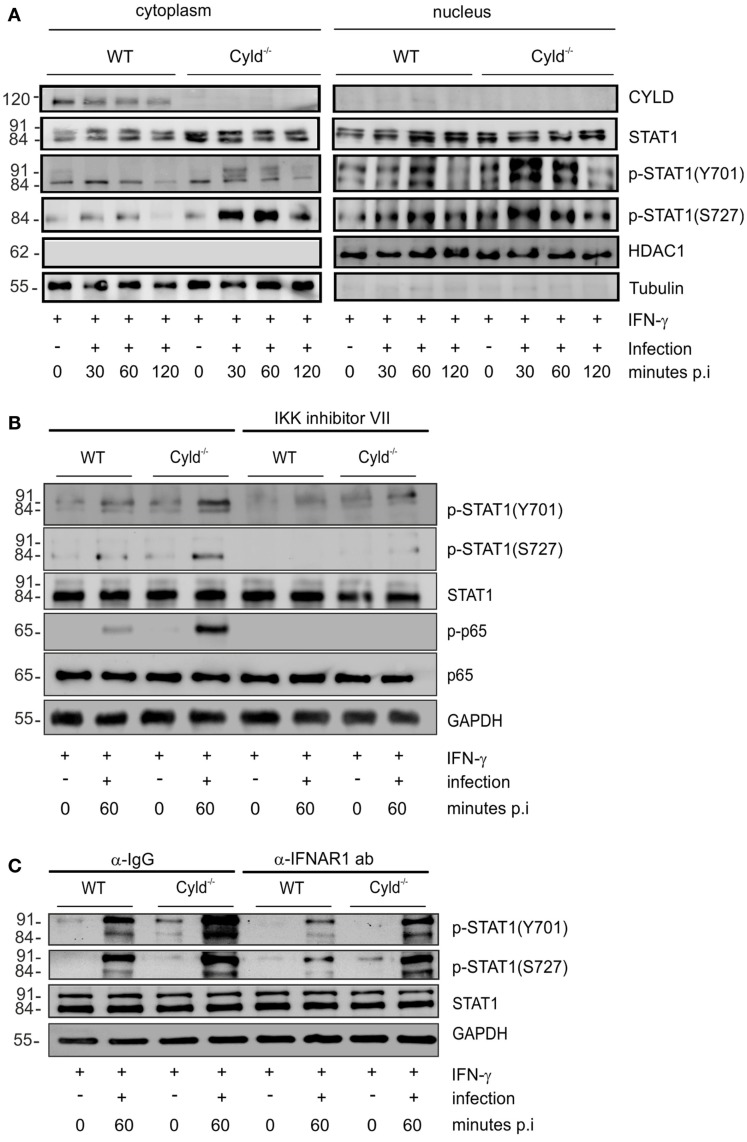
**CYLD diminishes activation of STAT1 upon stimulation with IFN-γ**. **(A–C)** 1 × 10^6^ WT and Cyld^−/−^ BMDM were stimulated with IFN-γ (100 U/ml) for 24 h. Thereafter, the indicated groups of BMDM were additionally infected with Lm (MOI 5:1) for 24 h. Proteins were isolated from uninfected (0 min p.i.) and infected (30, 60 min p.i.) BMDM as indicated. **(A)** Proteins were isolated from the cytoplasm and nucleus of BMDM respectively at the indicated time points after infection. WBs were stained with anti-tubulin and anti-histone deacetylase (HDAC) as marker proteins for the cytoplasm and nucleus, respectively. In addition to total STAT1, phosphorylation of STAT1 at position Y701 and S727, respectively, was analyzed by WB. **(B)** BMDM were either left untreated or treated with IKK inhibitor (1 μM) beginning in parallel to IFN-γ treatment. Proteins were isolated at the indicated time points and WB analysis for p-65, p-p-65, STAT1, p-STAT1 (Y701), p-STAT1 (S727), and GAPDH was performed. **(C)** IFN-γ-stimulated, -uninfected, and -infected BMDM were either treated with control IgG or anti-IFNAR1 antibody (30 μg/ml) beginning 30 min before infection with Lm. Proteins were isolated at the indicated time points and WB analysis for p-65, p-p-65, STAT1, p-STAT1 (Y701), p-STAT1 (S727), and GAPDH was performed. In **(A–C)**, data represent one of the two independent experiments.

Mice with macrophage-specific deletion of STAT1 are susceptible to listeriosis (41). Since IFN-γ augments bactericidal activity and NO production of Lm-infected macrophages [Figure 1A (39, 40)] and the immunostimulatory activity of IFN-γ is mediated by the transcription factor STAT1, we studied the impact of CYLD on the activation of STAT1 in Lm-infected IFN-γ-stimulated BMDM. Within 30 min post infection, the phosphorylation of cytoplasmic and nuclear STAT1 (Y701) and STAT1 (S727) was increased in Cyld^−/−^ as compared to WT BMDM (Figure 2A). To study how CYLD reduces STAT1 activation and nuclear accumulation in IFN-γ-stimulated Lm-infected BMDM, we analyzed whether CYLD might directly bind to STAT1. However, immunoprecipitation experiments showed that CYLD and STAT1 did not interact with each other (data not shown). Importantly, the inhibition of NF-κB activity by an IKK inhibitor decreased p-STAT1 to equally low levels in both Lm-infected WT and Cyld^−/−^ BMDM indicating that the activation of STAT1 was dependent on the augmented *Listeria*-induced NF-κB activation (Figure 2B).

In listeriosis, type I IFNs, which also activate STAT1, are mainly produced by macrophages and exacerbate the diseases (5–7). Therefore, we studied the effect of type I IFNs on the CYLD-mediated suppression of STAT1 activation in Lm-infected IFN-γ-stimulated BMDM. Blocking of IFNAR1 by IFNAR1-specific antibodies in Lm-infected IFN-γ-stimulated BMDM decreased p-STAT1 (Y701) and p-STAT1 (S727) levels in Lm-infected WT and Cyld^−/−^ BMDM without abolishing the strongly enhanced STAT1 activation of Cyld^−/−^ BMDM (Figure 2C). Thus, in addition to IFN-γ, the activation of STAT1 was partially dependent on autocrine type I IFN-mediated activation of STAT1 (Figure 2C).

### CYLD Impairs RIPK2-Mediated Activation and Antibacterial Function of BMDM

**Figure 3:**
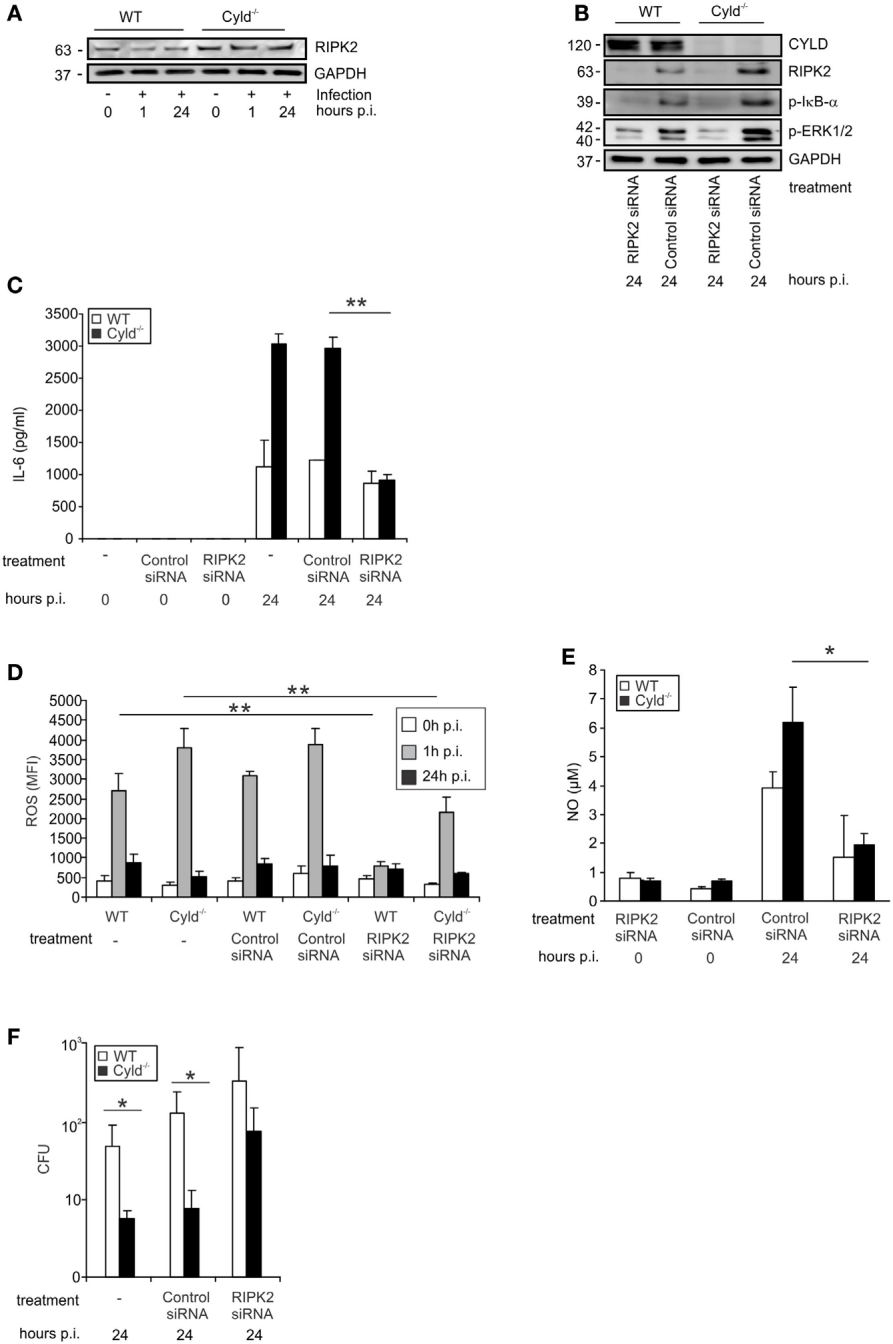
**CYLD-mediated inhibition of NF-κB and MAP kinases activation is dependent on RIPK2**. WT and Cyld^−/−^ BMDM were cultivated and the indicated subgroups of BMDM (1 × 10^6^/well) were transfected with RIPK2 and control siRNA, respectively, beginning 48 h before stimulation with IFN-γ (100 U/ml, all groups of BMDM). After 24 h of IFN-γ-stimulation, the indicated groups of BMDM were infected with Lm (MOI 5:1) for the indicated time points or left untreated (0 h p.i.). **(A)** Proteins were isolated from indicated groups of 1 × 10^6^ uninfected and Lm-infected BMDM and analyzed for RIPK2 and GAPDH protein expression by WB. In this experiment, the same proteins were used as in Figure 1F. **(B)** BMDMs were treated with siRNA and infected with Lm as indicated. Proteins were isolated and analyzed by WB for RIPK2, p-ERK1/2, p-IκBα, and GAPDH protein expression. **(C)** The indicated groups of RIPK2 and control siRNA-treated WT and Cyld^−/−^ BMDM were infected with Lm or left uninfected. The supernatant of BMDM cultures was harvested before infection (0) or 24 h post infection (p.i.). IL-6 was determined in the supernatant by flow cytometry using cytometric bead array. Data show the mean + SD of triplicate wells per group. **(D)** BMDMs were transfected with RIPK2 and control siRNA and infected with Lm as indicated. Intracellular ROS production of 1 × 10^6^ WT and Cyld^−/−^ BMDM was analyzed by flow cytometry employing a ROS detection kit. BMDM were analyzed before infection (0 h p.i.) and at the time point of infection (1 h p.i. and 24 h p.i.). Data show the mean + SD of triplicate wells per group. **(E)** The supernatant of the indicated groups of RIP2K and control siRNA treated, uninfected, and infected WT and Cyld^−/−^ BMDM (1 × 10^6^ cells) was harvested before and 24 h p.i. NO was determined photometrically using a Griess assay kit. Data show the mean + SD of triplicate wells per group. **(F)** Twenty-four hours p.i., the bacterial load was determined in Lm-infected WT and Cyld^−/−^ BMDM (1 × 10^6^ cells), which were treated with RIPK2 and control siRNA as indicated. Data show the mean + SD of triplicate wells per group. In **(A–F)**, data represent one of the two independent experiments with similar results.

Since RIPK2 activates the canonical NF-κB and ERK pathways, upon intracellular recognition of Lm by the NOD2 receptor, we studied the impact of CYLD on RIPK2 expression in IFN-γ-stimulated Lm-infected BMDM. In IFN-γ-stimulated and Lm-infected BMDM, RIPK2 protein was increased in Cyld^−/−^ as compared to WT BMDM (Figure 3A). To study whether increased RIPK2 levels in Cyld^−/−^ BMDM led to the increased activation of NF-κB and MAPKs pathways, IFN-γ-stimulated WT and Cyld^−/−^ BMDM were treated with RIPK2 and control siRNA, respectively, for 48 h prior to Lm infection. WB analysis showed an efficient knockdown of RIPK2 in RIPK2-siRNA-treated BMDM 24 h p.i., whereas treatment with control siRNA did not influence RIPK2 protein levels (Figure 3B). RIPK2 knockdown caused a strong decrease of p-IκB-α (Figure 3B) and p-ERK (Figure 3B) and diminished differences in the expression levels of both molecules in Cyld^−/−^ and WT BMDM. In addition, RIPK2-siRNA-treatment of IFN-γ-stimulated Lm-infected BMDM significantly reduced IL-6 (Figure 3C), ROS (Figure 3D), and NO levels (Figure 3E) in both Cyld^−/−^ and WT BMDM. Consequently, siRNA-mediated RIPK2 inhibition significantly increased colony-forming units (CFUs) in RIPK2-siRNA-treated Cyld^−/−^ and WT BMDM (Figure 3F). Thus, the improved immune response in Lm-infected Cyld^−/−^ BMDM is RIPK2 dependent.

### K63 Deubiquitination of RIPK2 by CYLD

**Figure 4:**
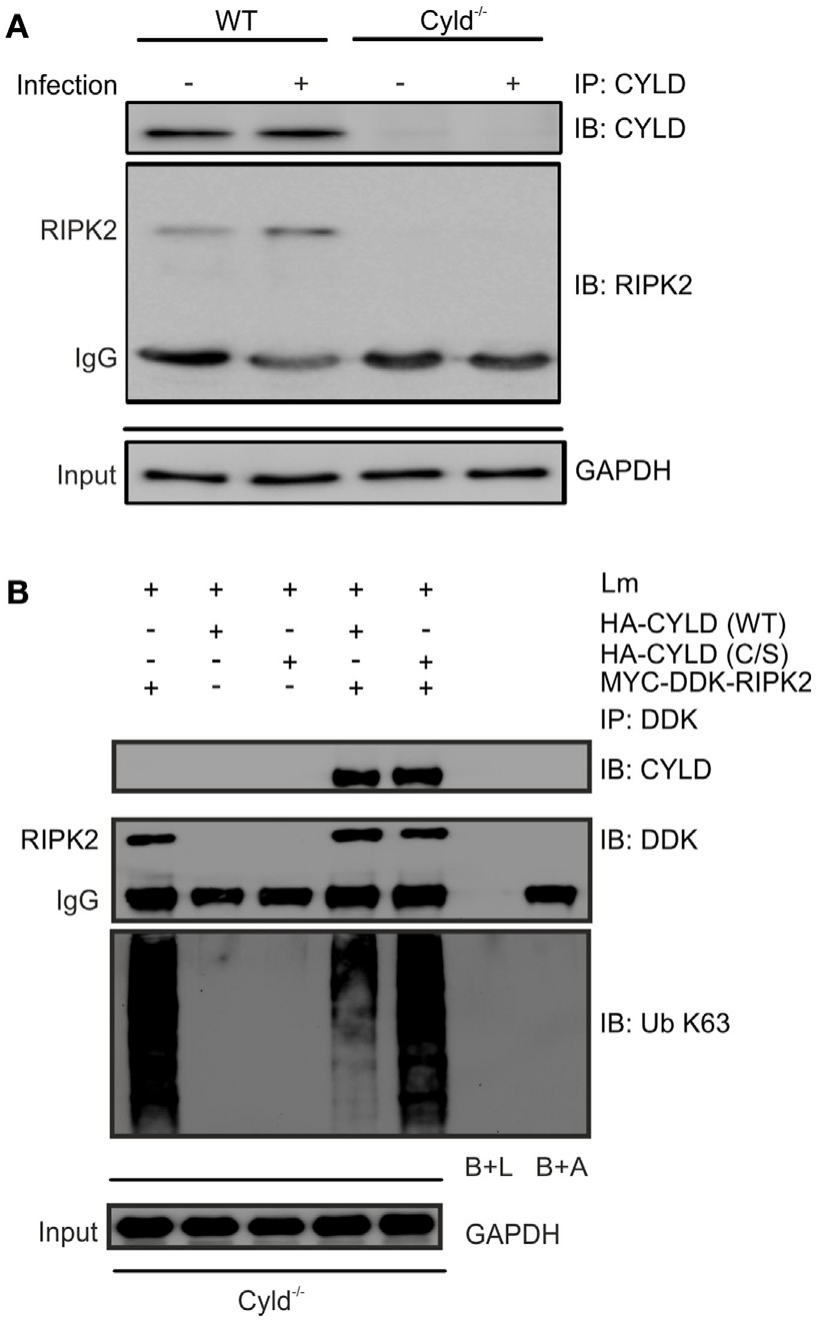
**CYLD cleaves K63-polyubiquitin chains from RIPK2**. **(A)** Proteins from 1 × 10^6^ IFN-γ stimulated, infected (MOI 5:1, 24 h after infection) and uninfected WT and Cyld^−/−^ BMDM were immunoprecipitated with CYLD antibody. Immunoprecipitates were stained for CYLD and RIPK2. GAPDH was used as loading control. **(B)** Cyld^−/−^ BMDM (1 × 10^6^ cells) were transfected with MYC-DDK-RIPK2, HA-CYLD (WT), and mutant HA-CYLD (C/S), which lacks catalytic activity, as indicated. After 24 h of infection, total lysates were immunoprecipitated with anti-DDK. Immunoprecipitates were stained for the indicated proteins. Beads plus lysate without antibody before immunoprecipitation (B + L), beads plus DDK antibody without lysate (B + A), and GAPDH were used as control. In **(A,B)**, data represent one of the two independent experiments.

To study whether CYLD directly regulates RIPK2 in Lm-infected BMDM, we immunoprecipitated CYLD from Lm-infected IFN-γ-stimulated WT and Cyld^−/−^ BMDM. WB analysis of immunoprecipitates detected CYLD and RIPK2 only in WT BMDM but not in Cyld^−/−^ BMDM indicating that CYLD interacts with RIPK2 (Figure 4A). Transfection of Cyld^−/−^ BMDM with MYC-DDK-RIPK2, HA-CYLD (CYLD WT), and catalytically inactive CYLD (CYLD C/S) followed by immunoprecipitation of DDK-RIPK2 further showed that WT CYLD reduced K63 ubiquitination of RIPK2 (Figure 4B). Catalytically inactive CYLD interacted with RIPK2 but failed to reduce K63 ubiquitination of RIPK2 (Figure 4B).

### CYLD Regulates ERK1/2-Mediated Production of Antibacterial Effector Molecules, Autophagy, and Control of Lm

**Figure 5:**
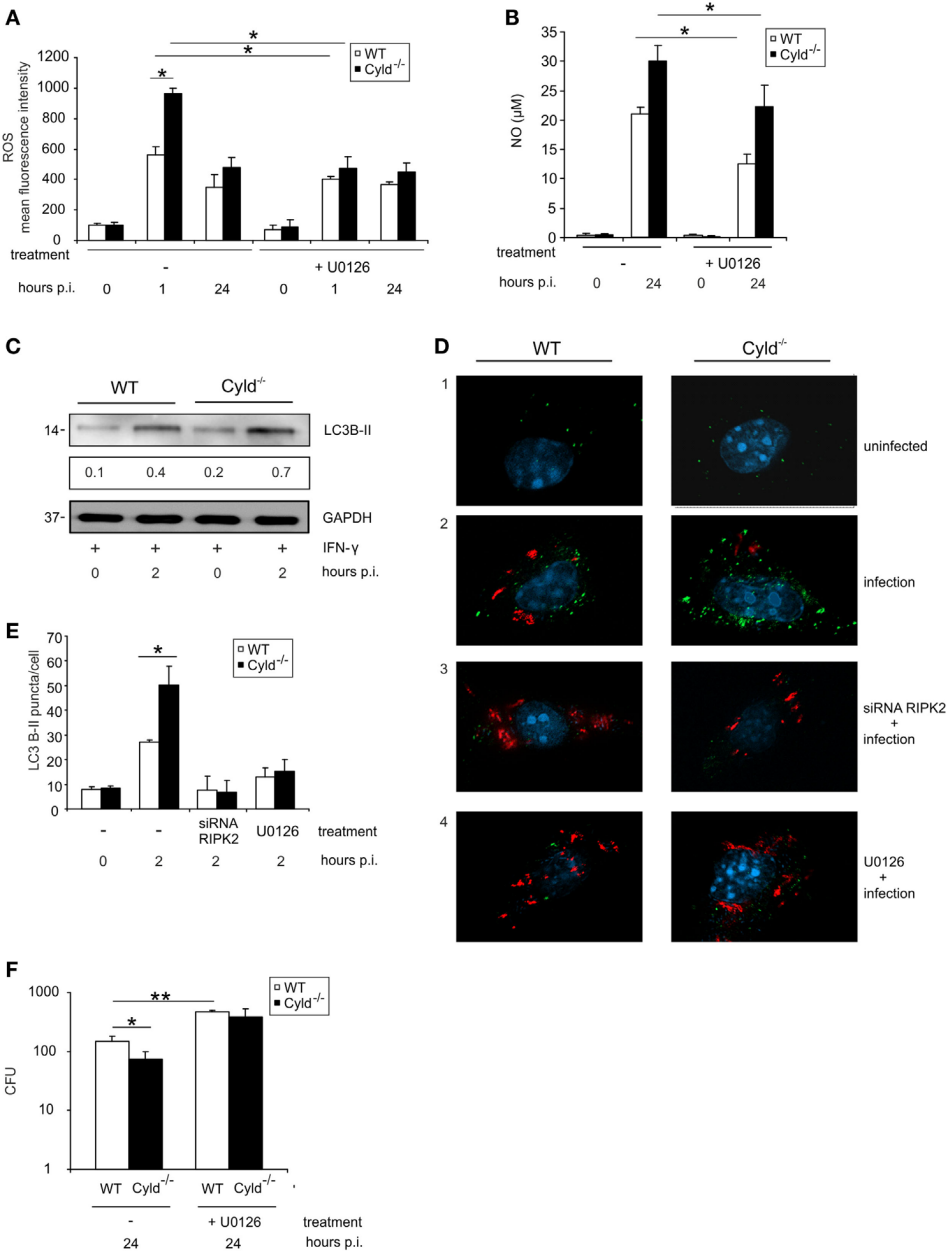
**ERK-dependent production of anti-listerial effector molecules and autophagy protects against *Listeria* infection of BMDM**. WT and Cyld^−/−^ BMDM (1 × 10^6^/well) were cultivated and stimulated with IFN-γ (100 U/ml) for 24 h. Thereafter, the indicated groups of BMDM were infected with Lm (MOI 5:1) or left uninfected (0 h p.i.). **(A)** IFN-γ-stimulated BMDM were either untreated or treated with the ERK inhibitor U0126 (1 μM) beginning 2 h before infection. Intracellular ROS was determined by flow cytometry using a ROS detection kit at the indicated time points. Data show the mean + SD of triplicate wells per group. **(B)** Uninfected and infected, IFN-γ-stimulated WT, and Cyld^−/−^ BMDM were treated with the ERK inhibitor U0126 (1 μM) beginning 2 h before infection as indicated. The supernatant was harvested before and 24 h p.i. NO was determined photometrically in the supernatant using a Griess assay kit. Data show the mean + SD of triplicate wells per group. **(C)** Proteins were isolated from IFN-γ-stimulated, uninfected (0 h p.i.), and infected (2 h p.i.) WT and Cyld^−/−^ BMDM and WB analysis for the autophagosomal marker LC3B-II and GAPDH was performed. LC3B-II expression was determined using a densitometer and data show the mean of three WB performed with independent samples. **(D)** Immunofluorescence images from uninfected and Lm-infected WT and Cyld^−/−^ BMDM are shown. Cells (5 × 10^5^ BMDM per slide) were either untreated or treated with RIPK2 siRNA (D3, beginning 48 h before infection) or the ERK inhibitor U0126 (D4, 1 μM, beginning 2 h before infection). BMDM were stained for *Listeria* (red), LC3B-II (green), and the nucleus (DAPI, blue). **(E)** Two hours p.i., the number of LC3B-II puncta per cell from the experimental groups shown in **(D)** was determined microscopically. Data show the mean + SD of three slides and 100 BMDM/slide per group. **(F)** CFUs were determined in 1 × 10^6^ Lm-infected WT and Cyld^−/−^ BMDM, which were either untreated or treated with ERK inhibitor U0126 (10 μM) beginning 2 h before infection. Data show the mean + SD of triplicate wells per group. In **(A–F)**, data represent one of the two independent experiments.

Since NOD2-mediated RIPK2 activation results in the activation of ERK (Figure 1F), we further studied the impact of CYLD on ERK1/2-dependent pathogen control and production of ROS and NO. Inhibition of ERK by pretreatment of BMDM with U0126, an ERK1/2 inhibitor, increased CFU and reduced the levels of ROS (Figure 5A) as well as NO (Figure 5B) in BMDM from both mouse strains. Whereas ERK1/2 inhibition abolished differences between WT and Cyld^−/−^ mice with respect to CFU and ROS, NO levels were still significantly increased in Cyld^−/−^ mice.

Autophagy is an intrinsic defense mechanism against Lm and, therefore, we further investigated whether CYLD-dependent RIPK2 and ERK1/2 activation influences the formation of autophagosomes. WB for the autophagosome marker LC3B-II indicated increased autophagosome formation in Cyld^−/−^ BMDM (Figure 5C). Fluorescence microscopy results showed increased number of autophagosomes (green) (Figures 5D,E) in Cyld^−/−^ BMDM accompanied by reduced Lm burden (red) (Figure 5D). Furthermore, the enhanced formation of autophagosome in Cyld^−/−^ BMDM was abolished upon knockdown of RIPK2 by siRNA (Figures 5D,E) as well as inhibition of the downstream molecule ERK1/2 (Figures 5D,E). The reduction in autophagomes resulted in increased Lm load in BMDM from both groups (Figure 5D) indicating that CYLD inhibits the formation of autophagosomes by regulating RIPK2-mediated ERK1/2 expression. In accordance to the reduced production of ROS, NO, and autophagosomal killing of Lm in ERK-inhibitor-treated WT and Cyld^−/−^ BMDM, inhibition of ERK resulted in significantly increased CFUs in both groups (Figure 5F).

## Discussion

The present study identified CYLD as a novel and important inhibitor of RIPK2-dependent protective immune responses in Lm-infected murine macrophages. Activation of RIPK2 by the Lm-sensing intracellular pattern-recognition receptor NOD2 is essential for the downstream stimulation of the NF-κB and MAPK pathways (42, 43), NOD2-induced immune responses in macrophages (25) and the control of listeriosis *in vivo* (17). Previous studies have shown that CYLD limited RIPK2-mediated activation of NF-κB by removing K63-polyubiquitin chains from NEMO (8, 44). In addition to NEMO, the proinflammatory function of RIPK2 is also dependent on its K63 polyubiquitination. Although multiple E3 ligases including Pellino-3, ITCH, cellular inhibitor of apoptosis (cIAP)-1, cIAP2, X-linked apoptosis of protein (XIAP) and TRAF2, TRAF5, and TRAF6 (9, 11–13, 45) perform K63 polyubiquitination of RIPK2, the only counterregulatory RIPK2 K63-deubiquitinating enzyme identified so far is A20 (10). Here, we demonstrate that CYLD also directly binds to RIPK2 and cleaves K63-polyubiquitin chains from RIPK2 in Lm-infected macrophages resulting in a reduced activation of NF-κB, p38 MAPK, and ERK1/2 and, consequently, in an impaired control of Lm in macrophages.

In addition to listeriosis, RIPK2 plays essential protective role upon infection with the intracellular bacteria *Legionella pneumophila*, *Chlamydophila pneumonia*, and *Mycobacterium tuberculosis* (15, 17, 46). In sharp contrast, RIPK2 promotes inflammation and lethality during infection with the Gram-negative bacteria *Pseudomonas aeruginosa* and *E. coli* due to excessive activation of NF-κB and MAPK (47). Interestingly, Cyld deficiency also increases susceptibility to infection with *E. coli* and *H. influenzae* due to immunopathology induced by the hyperactivation of the same signaling pathways (37, 38), whereas it protects from lethal listeriosis (36). Thus, the underlying infection determines the impact of RIPK2 and CYLD on the outcome of the disease and our identification of a direct inhibition of RIPK2 by CYLD provides a mechanistical explanation of the antagonistic effects of these two signaling molecules.

The crucial importance of the CYLD/RIPK2 interaction for the reduction of protective anti-Lm immune responses is shown by the complete abolishment of the protective effect of Cyld deficiency by RIPK2 inhibition. In the absence of RIPK2, Lm-infected WT, and Cyld^−/−^ macrophages produced less ROS and NO, showed reduced autophagy and, finally, exhibited diminished control of the pathogen without any significant differences between Cyld-competent and -deficient BMDM.

CYLD regulates the NF-κB-dependent production of antibacterial ROS and NO in macrophages (36, 48). Here, we also demonstrate that CYLD also inhibits ERK1/2-dependent ROS production. However, compared to NF-κB-dependent ROS production, the effect of ERK1/2 inhibition on reduction of ROS was lower (≈50%) in both WT and Cyld^−/−^ BMDM. In addition, NO production was significantly reduced by ERK1/2 inhibition in both Lm-infected WT and Cyld^−/−^ BMDM. In contrast to ROS, ERK inhibition did not abolish the increased production of NO in Cyld^−/−^ BMDM. Thus, both the increased NF-κB and ERK1/2 activation in Cyld^−/−^ BMDM contributes to the increased ROS and NO production, although NF-κB seems to be the more potent regulator. This is also illustrated by the more than 100-fold increase of CFU in both NF-κB inhibited WT and Cyld^−/−^ BMDM as compared to a <10-fold increase in macrophages with ERK1/2 inhibition (Figure 5F) (36).

Autophagy is an essential cell intrinsic mechanism to combat intracellular *Listeria* and RIPK2 is known to regulate autophagy via ERK activation (25). Here, we identify for the first time that CYLD also impairs autophagosome formation in Lm-infected macrophages (Figures 5D–E). Inhibition of RIPK2 and ERK caused a reduction in autophagosome formation accompanied by increased Lm burden in both WT and Cyld^−/−^ BMDM. Thus, CYLD impairs production of antibacterial effector molecules and autophagosome formation, which all contribute to the impaired pathogen control.

The convergence of type I and type II IFN signaling on the level of STAT1 places this signaling molecule in a central position in antibacterial defense of macrophages (41).

In good agreement with previous studies (39, 40), IFN-γ prestimulation of macrophages greatly augmented their capacity to control Lm. In extension and importantly, the protective effect of Cyld deficiency on the control of Lm was completely dependent on IFN-γ prestimulation of macrophages. IFN-γ prestimulation of WT and Cyld^−/−^ BMDM without infection resulted in an equally weak activation and nuclear translocation of STAT1, a critical IFN-γ-induced transcription factor (Figure 2A). In accordance with the CYLD-independent activation of STAT1 by IFN-γ, we could not detect a direct interaction of CYLD with STAT1. In sharp contrast, STAT1 activation was much stronger in Cyld^−/−^ BMDM upon additional Lm infection. This suggests an indirect regulation of STAT1 activation by NOD2/RIPK2-dependent immune responses. In fact, we could identify that inhibition of NF-κB abolished the increased STAT1 activation in Lm-infected IFN-γ-stimulated Cyld^−/−^ BMDM (Figure 2B). This indirect regulation of STAT1 signaling by CYLD resembles the function of the deubiquitinating enzyme A20, which also inhibits STAT1 activation indirectly by suppressing NF-κB activation (49).

In addition to type II IFN, type I IFNs play an important role in the macrophage-mediated immune responses. The convergence of type I and type II IFN signaling on the level of STAT1 places this signaling molecule in a central position in antibacterial defense of macrophages (41). Previously, it has been shown that type I IFNs synergistically activates with NF-κB an anti-listerial response in Lm-infected macrophages (50), in good agreement, inhibition of the IFNAR resulted in a reduced activation of STAT1 in both WT and Cyld^−/−^ macrophages (Figure 2C). Thus, activation of the NOD2/RIPK2/NF-κB pathway and endogenous production of type I IFN cooperatively regulated the activation of STAT1 and gene transcription of proinflammatory and antibacterial mediators.

Taken together, our study identifies CYLD as a new interaction partner and regulator of RIPK2 kinase signaling pathways and as an inhibitor of anti-listerial immune responses of macrophages. Before, we have shown that CYLD inhibits protective fibrin production by hepatocytes in listeriosis (36). Thus, CYLD impairs the outcome of the infection by inhibiting several non-redundant protective host responses. This places CYLD in an attractive position as a therapeutic target molecule in severe infections with Lm and, potentially, other intracellular bacteria. In addition, it will be important to develop conditional CYLD-deficient mice to decipher the importance of CYLD in individual cell types under infectious disease conditions.

## Materials and Methods

### Ethics Statement

All animal experiments were in compliance with the German animal protection law in a protocol approved by the Landesverwaltungsamt Sachsen-Anhalt (file number: 203.h-42502-2-901, University of Magdeburg).

### Animals

Age- and sex-matched animals were used for the experiments. C57BL/6 WT were obtained from Janvier (Le Genest Saint Isle, France) and C57BL/6 Cyld^−/−^ mice were kindly provided by Dr. Ramin Massoumi (Department of Laboratory Medicine, Malmö, Sweden) (34). All animals were kept under conventional conditions in an isolation facility of the Otto-von-Guericke University Magdeburg. Experiments were approved and supervised by local governmental institutions.

### *Listeria* *monocytogenes*

*Listeria monocytogenes* (EGD strain) was grown in brain–heart infusion broth (Merck, Darmstadt, Germany) and aliquots of log-phase cultures were stored at −80°C. For infection of BMDM, fresh log phase cultures were prepared from frozen stock.

### Bone Marrow-Derived Macrophages

Freshly prepared BMDM were used for each experiment. Femur and tibia were aseptically removed from mice, the bone ends were cut off, and the bone marrow cells were flushed out using PBS (4–8°C; Gibco, Darmstadt, Germany). The cell suspension was washed twice with PBS by centrifugation (490 × *g*, 5 min) and cultured in Petri dishes (ø 85 mm, Sarstedt, Numbrecht, Germany) in Dulbecco’s modified Eagle’s medium with high glucose and l-glutamine (DMEM) supplemented with 10% FCS, 1% non-essential amino acids (all from PAA, Coelbe, Germany), 1% glutamine (Biochrom, Berlin, Germany), 50 μM 2-­mercaptoethanol (Gibco, Darmstadt, Germany), and 20 ng/ml macrophage colony-stimulating factor (M-CSF) (PeproTech, Hamburg, Germany) for 3 days. Medium was changed on day 3 and non-adherent cells were removed by washing with PBS. Adherent BMDM were harvested at day 6 and used for experiments. The purity of the cultured macrophages ranged from 95 to 99%, as determined by flow cytometry using the macrophage-specific markers CD11b and F4/80 (F4/80 eFluor450 (clone: BM8)) and CD11b APC (clone: M1/70; both eBioscience, Frankfurt, Germany).

### *In vitro* Infection of BMDM with Lm

About 1 × 10^6^ BMDM were stimulated in six-well plates (Greiner bio-one, Frickenhausen, Germany) with IFN-γ (100 U/ml) (PeproTech, Hamburg, Germany), a dose previously standardized by us (36) for 24 h. Thereafter, stimulated and unstimulated BMDM were infected with Lm at a multiplicity of infection (MOI) of 5:1 in DMEM supplemented with 10% FCS, 1% non-essential amino acids, 1% glutamine, 50 μM 2-mercaptoethanol, and 20 ng/ml M-CSF. For infection, Lm were thawed from frozen stocks (−80°C) and added to brain–heart infusion broth. The optical density of log-phase cultures were determined using a photometer (Eppendorf, Hamburg, Germany). Cultures with an optical density of 0.1, which corresponds to a dose of 1 × 10^8^ Lm/ml according to a previously established standard growth curve, were pelleted by centrifugation (870 × *g*, 4°C, 10 min) and the multiplicity of infection MOI was adjusted to 5:1 in DMEM supplemented with 10% FCS, 1% non-essential amino acids, 1% glutamine, and 50 μM 2-mercaptoethanol. In each experiment, the bacterial dose used for infection was controlled by plating an inoculum on brain–heart infusion agar and counting colonies after incubation at 37°C for 24 h.

After 1 h of infection (37°C, 5% CO_2_), 30 μg/ml gentamicin (Sigma-Aldrich) was added for additional 3 h to kill extracellular bacteria. Thereafter, infected BMDM were washed twice with PBS and further cultivated in DMEM supplemented with 15 μg/ml gentamicin for the indicated time points. For inhibition of NF-κB, BMDM were treated with IKK inhibitor VII (1 μM; Calbiochem, Darmstadt, Germany) beginning 24 h before infection (concentration and incubation time were pretested before usage). For the inhibition of ERK, BMDM were treated with the ERK inhibitor U0126 (1 μM; Calbiochem) beginning 2 h before infection according to the manufacturer’s recommendation. The inhibitors were dissolved in DMSO (5 mg U0126/ml; 10 mg IKK inhibitor VII/ml). The final concentration of DMSO used in the cultures was 0.1%. For the inhibition of IFNAR1, BMDM were pretreated with either anti-IFNAR1 or control IgG (30 μg/ml) 30 min before infection according to the manufacturer’s recommendation (BioLegend, Fell, Germany).

### Colony-Forming Units

At the indicated time points p.i., Lm-infected BMDM cultures were centrifuged (5 min, 490 × *g*) and the medium was discarded. Thereafter, the cells were washed with PBS to remove traces of remaining antibiotics. After centrifugation (5 min, 490 × *g*) and removal of PBS, the cell pellets were lysed in 0.1% Triton-X-100 and serial dilutions were plated on brain–heart infusion agar in Petri dishes (ø 85 mm; Merck, Darmstadt, Germany). Bacterial colonies were counted microscopically using a grid plate after incubation at 37°C for 24 and 48 h. The counting was conducted blindly and 16 fields were counted.

### Protein Isolation and Western Blot

Interferon gamma-stimulated Lm-infected BMDM were washed in PBS and resuspended in 4–8°C cold lysis buffer containing 50 mM Tris–HCl (pH 7.4), 5 mM EDTA, 100 mM NaCl, 1% Triton-X-100, 10% glycerol, 10 mM KH_2_PO_4_, 0.5% Na-deoxycholate, 1 mM phenylmethanesulfonyl fluoride 1 mM NaF, 1 mM Na_4_O_7_P_2_, 1 mM Na_3_VO_4_ and aprotinin, leupeptin, and pepstatin (1 μg/ml each) (all reagents from Sigma, Taufkirchen, Germany) for 30 min and centrifuged (19,000 × *g*, 4°C, 10 min). The supernatant was collected and the protein concentration was determined by a commercial protein assay kit (Bio-RAD, Munich, Germany). A 1× lane marker reducing sample buffer (Thermo Scientific, Dreieich, Germany) was added to the samples and proteins were denatured at 99°C for 5 min. Equal amounts of proteins were separated on SDS-polyacrylamide gels (6–12%) and transferred semidry (220 mA, 60 min) on polyvinylidene fluoride membranes, preactivated in methanol. Unspecific binding of antibodies was blocked by incubating the membranes with either Blotto B [1% milk powder plus 1% bovine serum albumin (BSA)], 5% milk powder, or 5% BSA for 1 h. The proteins were stained for GAPDH, STAT-1, phospho-STAT1 S727, phospho-STAT1 Y701, phospho-p65, p65, phospho-p38, p38, phospho-IκBα, phospho-ERK1/2, ERK1/2, CYLD, LC3B, ubiquitin, K63-linkage-specific polyubiquitin (all antibodies from Cell Signaling Technology, Frankfurt, Germany), RIPK2 (Abcam, Cambridge, UK), tubulin (Sigma-Aldrich, Steinheim, Germany), and HDAC1 (Santa Cruz, Heidelberg, Germany). Primary antibodies were used at a dilution of 1:1000 in specific blocking medium as recommended by the supplier. Following overnight incubation, membranes were washed three times using Tris-buffered saline with 0.1% Tween 20 (TBST) for 10 min each and further incubated with 1:1000 diluted anti-mouse or anti-rabbit secondary antibodies (Dako, Hamburg, Germany) for 1 h. The blots were washed for three times in TBST 10 min each and developed using an ECL Plus kit (GE Healthcare, Freiburg, Germany). For quantification of protein intensities by densitometry, WB images were captured using the Intas Chemo Cam Luminescent Image Analysis system^®^ (INTAS Science Imaging Instruments, Göttingen, Germany) and analyzed with the LabImage 1D software^®^ (Kapelan Bio-Imaging Solutions, Leipzig, Germany).

### Transfection of BMDM

Bone marrow-derived macrophages were transiently transfected with hemagglutinin (HA)-CYLD WT, HA-CYLD C/S (catalytically inactive CYLD), and MYC-DDK RIPK2 (Origene, Rockville, MD, USA) plasmids, respectively, as indicated using Neon transfection system (Invitrogen, Darmstadt, Germany). PBS (without Ca^2+^ and Mg^2+^) was added to 1 × 10^7^ BMDM followed by centrifugation (5 min, 490 × *g*) and removal of the supernatant. The cell pellet was resuspended in 1 ml of resuspension buffer R (provided in the Neon transfection system kit). The transfection was performed according to the manufacturer’s instructions using a single pulse of 1000 V for 20 ms. The transfected BMDM were plated in six-well plates (Greiner bio-one, Frickenhausen, Germany) containing culture medium (DMEM supplemented with 10% FCS, 1% non-essential amino acids, 1% glutamine, 50 μM 2-mercaptoethanol, and 20 ng M-CSF/ml) and incubated at 37°C for 24 h before using them for experiments. The efficiency of transfection was controlled by WB for the indicated proteins.

### Immunoprecipitation

Uninfected and IFN-γ-stimulated Lm-infected BMDM as well as Cyld^−/−^ BMDM transfected with MYC-DDK-RIPK2, HA-CYLD (WT), and mutant HA-CYLD (C/S) were lysed on ice using the same protein lysis buffer as described above in “Protein isolation and Western Blot.” In a preclearing phase, Gamma Bind™ G Sepharose™ beads (GE Healthcare Europe GmbH, Freiburg Germany) were incubated with cell lysates under continuous shaking at 4°C for 30 min. The beads were removed by centrifugation (10 min, 10,000 × *g*, 4°C) and equal amounts of lysates were incubated with anti-RIPK2 (1:100) and anti-CYLD (1:200) antibodies, respectively, at 4°C overnight. The immune complexes were captured by incubating with fresh Gamma Bind™ G Sepharose™ beads at 4°C overnight. The beads were then washed three times with PBS by pulse centrifugation (1000 × *g*, 30 s). The pellet containing the Gamma Bind™ G Sepharose™ immune complexes was resuspended in 1× lane marker reducing sample buffer and boiled at 99°C for 5 min. Thereafter, samples were centrifuged (1000 × *g*, 30 s), the supernatant was collected and used to detect RIPK2, CYLD, K63 ubiquitin, by WB. GAPDH was used as the input control.

### Measurement of NO

The concentration of NO in the cell culture medium was measured using a Griess Assay Kit (Promega, Mannheim, Germany) according to the manufacturer’s instructions. In brief, the cells were centrifuged (490 × *g*, for 5 min) to harvest the supernatant. Triplicates of diluted standard and supernatant (50 μl each) were added to the wells of a 96-well flat-bottom plate (Greiner bio-one, Frickenhausen, Germany). Subsequently, 50 μl of sulfanilamide solution was dispensed to the standard and the experimental samples and incubated in the dark for 10 min. Thereafter, 50 μl of *N*-(1-naphthyl) ethylenediamine solution was added to all samples, which were further incubated in the dark for 10 min. The NO concentration was determined by measuring samples at 540 nm using a Synergy^®^ microplate reader (Biotek, Berlin, Germany) within 30 min.

### ROS/RNI Detection

The intracellular production of reactive oxygen and nitrogen species (ROS/RNI) was determined with a total ROS detection kit (Enzo Life Science, Lörrach, Germany). In brief, 1 × 10^6^ freshly prepared BMDM were infected with Lm at an MOI of 5:1. After infection, the cells were centrifuged (490 × *g*, for 5 min), the supernatant was discarded and the cell pellet was washed twice with 2 ml of 1× washing buffer. Thereafter, the cell pellet was resuspended in 500 μl detection solution (500 μl 1× washing buffer + 0.1 μl detection reagent) and incubated in the dark at 37°C for 30 min. BMDM stimulated with pyocyanin for 30 min were used as a positive control. BMDM stimulated with *N*-acetyl-l-cystein for 1 h were used as the negative control. The samples were analyzed by flow cytometry using a FACS Canto II (BD, Heidelberg, Germany). Enhanced ROS production was detected as an increase in the mean fluorescence (490/525 nm) of the samples over the control.

### Cytometric Bead Array

The level of IL-6 and IL-12/IL-23/p40 in the supernatant of the BMDM cell cultures were analyzed by flow cytometry using the cytometric bead array (CBA) from BD Biosciences (Heidelberg, Germany) using the manufacturer’s protocol. The cells were centrifuged (490 × *g*, for 5 min) to harvest the supernatant. Cytokine concentration in the supernatant was determined by adding 50 μl of cytokine-specific capture bead mixture to 50 μl of supernatant and standard tubes. Phycoerythrin (PE) detection agent (50 μl) was added to each sample and incubated in the dark at 4°C for 2 h. The samples were washed with 1 ml washing buffer, centrifuged at 200 × *g* for 5 min and, thereafter, resuspended in washing buffer (300 μl). The cytokine levels were measured using a FACS Canto II (BD, Heidelberg, Germany) and analyzed with FCAP Array™ software (version 3, BD Biosciences).

### *In vitro* siRNA Treatment

About 30 pmol siRNA [predesigned RIPK2 siRNA or silencer negative control siRNA from Ambion (CA, USA)] and 18 μl of Lipofectamine^®^ RNAiMAX (Invitrogen, Darmstadt, Germany) were diluted separately in 150 μl Opti-MEM serum-free medium each. The siRNA and Lipofectamine were mixed at a ratio of 1:1 and incubated for 5 min at room temperature. A total of 250 μl of the siRNA–Lipofectamine mixture was added to each well in a six-well plate containing 1 × 10^6^ BMDM and incubated at 37°C for 48 h. The efficiency of siRNA-mediated RIPK2 silencing was controlled by WB.

### Immunofluorescence

About 5 × 10^5^ BMDM were cultured on flamed coverslips in 12-well plates (Greiner bio-one, Frickenhausen, Germany). BMDM were infected with Lm as described earlier. At the indicated time points after infection, the coverslips were fixed in 4% paraformaldehyde (Roth, Karlsruhe, Germany) for 10 min and washed three times with PBS for 10 min each. The cells were then permeabilized in 0.1% Triton-X-100 solution for 7 min and blocked in 10% goat serum, 0, 5% BSA, 0.3% Triton-X-100, and 5% sucrose for 45 min. For staining, mouse anti-Lm antibody (BioRad, Munich, Germany) and rabbit anti-mouse LC3B antibody (Cell Signaling Technology, Frankfurt, Germany) were used at dilution of 1:200 in blocking medium and incubated with constant shaking in the dark overnight. The coverslips were then washed three times with PBS for 10 min each and incubated with 1:200 diluted Alexa Flour^®^ 488 goat anti-rabbit and Alexa Flour^®^ 594 goat anti-mouse antibody, respectively (both from Invitrogen, Darmstadt, Germany) at room temperature for 1 h. Thereafter, the coverslips were washed three times with PBS for 10 min each. The coverslips were air-dried and mounted on ethanol-cleaned slides with ProLong^®^ Gold antifade reagent containing DAPI (Invitrogen) and the immunofluorescence was captured using Zeiss observer Z.1 microscope (Carl Zeiss GmbH, Oberkochen, Germany).

### Statistics

Statistical significance was determined using the two-tailed Student’s *t*-test or non-parametric Mann–Whitney rank-sum test. All experiments were performed at least twice. *p* Values of <0.05 were considered significant.

## Conflict of Interest Statement

The authors declare that the research was conducted in the absence of any commercial or financial relationships that could be construed as a potential conflict of interest.
